# Synapse-Specific Regulation Revealed at Single Synapses Is Concealed When Recording Multiple Synapses

**DOI:** 10.3389/fncel.2017.00367

**Published:** 2017-11-23

**Authors:** Justin Lines, Ana Covelo, Ricardo Gómez, Lan Liu, Alfonso Araque

**Affiliations:** ^1^Department of Neuroscience, University of Minnesota, Minneapolis, MN, United States; ^2^Instituto Cajal, Consejo Superior de Investigaciones Científicas, Madrid, Spain; ^3^Department of Statistics, University of Minnesota, Minneapolis, MN, United States

**Keywords:** astrocytes, endocannabinoids, synapse, synaptic efficacy, synaptic plasticity, minimal stimulation, synapse specific

## Abstract

Synaptic transmission and its activity-dependent modulation, known as synaptic plasticity, are fundamental processes in nervous system function. Neurons may receive thousands of synaptic contacts, but synaptic regulation may occur only at individual or discrete subsets of synapses, which may have important consequences on the spatial extension of the modulation of synaptic information. Moreover, while several electrophysiological methods are used to assess synaptic transmission at different levels of observation, i.e., through local field potential and individual whole-cell recordings, their experimental limitations to detect synapse-specific modulation is poorly defined. We have investigated how well-known synapse-specific short-term plasticity, where some synapses are regulated and others left unregulated, mediated by astrocytes and endocannabinoid (eCB) signaling can be assessed at different observational levels. Using hippocampal slices, we have combined local field potential and whole-cell recordings of CA3-CA1 synaptic activity evoked by Schaffer collateral stimulation of either multiple or single synapses through bulk or minimal stimulation, respectively, to test the ability to detect short-term synaptic changes induced by eCB signaling. We also developed a mathematical model assuming a bimodal distribution of regulated and unregulated synapses based on realistic experimental data to simulate physiological results and to predict the experimental requirements of the different recording methods to detect discrete changes in subsets of synapses. We show that eCB-induced depolarization-induced suppression of excitation (DSE) and astrocyte-mediated synaptic potentiation can be observed when monitoring single or few synapses, but are statistically concealed when recording the activity of a large number of synapses. These results indicate that the electrophysiological methodology is critical to properly assess synaptic changes occurring in subsets of synapses, and they suggest that relevant synapse-specific regulatory phenomena may be experimentally undetected but may have important implications in the spatial extension of synaptic plasticity phenomena.

## Introduction

Synaptic transmission is a specialized form of communication between sensory cells, neurons and effector cells. Synaptic efficacy refers to the strength of communication between neurons, and mainly depends on the probability and amount of neurotransmitter released from presynaptic neurons and the number of postsynaptic receptors activated. Therefore, three parameters define synaptic transmission properties in a single synapse: the probability of neurotransmitter release, the synaptic potency (i.e., the number of postsynaptic receptors activated), and the synaptic efficacy that results from the combination of them (del Castillo and Katz, 1954; Hessler et al., 1993; Dobrunz and Stevens, 1997; Atwood and Karunanithi, 2002). These synaptic parameters can undergo activity-dependent dynamic changes, a phenomenon termed synaptic plasticity, that can be manifested with different temporal ranges, such as short- or long-term plasticity (Bliss and Collingridge, 1993; Stevens and Wang, 1994; Isaac et al., 1996; Dobrunz and Stevens, 1997; Perea and Araque, 2007; Navarrete and Araque, 2010; Navarrete et al., 2012) and have important consequences on brain function. For example, long-term plasticity is thought to represent the cellular basis of learning and memory processes (Morris et al., 1986; Bliss and Collingridge, 1993; Malenka and Nicoll, 1999). In each synapse, synaptic efficacy serves to integrate the information coming from different inputs, which can be regulated by neurotransmitters and neuromodulators (Scanziani et al., 1998). The level of fidelity in assessing synaptic transmission properties and the regulatory processes is therefore crucial for the proper appraisal of changes in synaptic efficacy to identify physiologically relevant processes.

Several electrophysiological methods are widely used to monitor synaptic transmission. For simplicity, we will refer to excitatory neurotransmission. Extracellular recordings of field excitatory postsynaptic potentials (fEPSP) combined with bulk stimulation of a large number of axons provide an estimation of synaptic transmission resulting from the near-simultaneous activity of multiple pre and postsynaptic neurons. Whole-cell patch-clamp recordings of excitatory postsynaptic currents (EPSC) evoked by bulk stimulation provide an estimate of the synaptic efficacy of multiple synaptic terminals into a single postsynaptic neuron. Finally, the minimal stimulation method, which activates a relatively limited number of axons, allows in certain brain areas (in which few presynaptic terminals originating from the same axon contact individual neurons) monitoring whole-cell recorded EPSCs resulting from the activity of a single synapse (Raastad, 1995; Isaac et al., 1996; Dobrunz and Stevens, 1997; Perea and Araque, 2007; Navarrete and Araque, 2010; Navarrete et al., 2012; Perea et al., 2016). Therefore, these three approaches assess synaptic transmission properties at different levels of observation.

Neuronal information has been proposed to be encoded across multiple synapses, requiring single cells to partake in countless representations at the synaptic scale (Buzsáki, 2010). In contrast to an overall adjustment of a population of synapses en-masse, precise changes must occur in particular subsets of synapses. These subtle changes in specific synapses could be concealed by the large number of synapses, but may nevertheless have important functional consequences. For example, certain neurotransmitters, such as GABA, may act on specific synapses within a cell, while leaving other synapses unaltered (Nusser et al., 2001). Likewise, astrocytes have been shown to exert subtle synapse-specific regulation of neurotransmission (Perea and Araque, 2007; Navarrete and Araque, 2010; Martín et al., 2015).

Endocannabinoid (eCB) signaling is known to regulate subsets of specific synapses through well-defined mechanisms (Wilson and Nicoll, 2001; Ohno-Shosaku et al., 2002; Brown et al., 2003; Chevaleyre and Castillo, 2004; Navarrete and Araque, 2010): eCBs released postsynaptically during neuronal activity are known to evoke: (1) a depolarization-induced suppression of excitation (DSE) by directly activating neuronal presynaptic cannabinoid receptors type 1 (CB1Rs; Ohno-Shosaku et al., 2002; Chevaleyre and Castillo, 2004); and (2) eCB-induced astrocyte-mediated lateral potentiation of synaptic transmission (eSP) by activating astrocyte CB1Rs, which leads to neuronal type I metabotropic glutamate receptor (mGluR) activation (Navarrete and Araque, 2010; Gómez-Gonzalo et al., 2015). Through these mechanisms, eCBs are known to modulate individual synapses, exerting their direct depressing action at relatively near synapses (≤60 μm) overcoming nearby astrocyte mediated potentiation, resigning observable potentiated effects to relatively distant synapses of the eCB source (Navarrete and Araque, 2010; Gómez-Gonzalo et al., 2015).

Determining the specific limitations to reliably detect synaptic changes with accurate fidelity is critical for the proper assessment of synaptic regulation. We combined experimental data obtained using the three electrophysiological methods of analysis of synaptic transmission parameters at different levels of observation (single synapses, synapse ensembles and large synapse populations) and mathematical modeling. Minimal stimulation activates a single fiber to observe the contained processing of a single synapse. Increasing fiber stimulation recruits synapse ensembles to study the aggregation of synapses in a cell, summing along dendritic arborization. Extracellular recording of bulk stimulation monitors a synapse population to assess the local environment. Using hippocampal slices, we stimulated Schaffer collaterals (SC) with either bulk or minimal stimulation, and recorded fEPSP in the CA1 region and EPSC in whole-cell recorded CA1 pyramidal neurons. We analyzed the synaptic changes induced by well-defined eCB-mediated phenomena, DSE and eSP. We found that both DSE and eSP could be reliably observed at the single synapse level, but were concealed when amalgamated with multiple synapses. Concealment was conserved at the synapse population level as well. Mathematical modeling relying solely on single synapse experimental data was able to recreate these findings and predict the theoretical sample size required to observe differences across multiple scales of observation.

## Materials and Methods

### Hippocampal Slice Preparation

All the procedures for handling and sacrificing animals were approved by the University of Minnesota Institutional Animal Care and Use Committee (IACUC) in compliance with the National Institutes of Health guidelines for the care and use of laboratory animals. Hippocampal slices were obtained from 12 to 21 days old C57BL/6J mice. Animals were anesthetized and decapitated, and the brain was rapidly removed and placed in ice-cold artificial cerebrospinal fluid (ACSF) containing (in mM): NaCl 124, KCl 5, NaH_2_PO_4_ 1.25, MgSO_4_ 2, NaHCO_3_ 26, CaCl_2_ 2 and glucose 10, and was gassed with 95% O_2_/5% CO_2_ (pH = 7.3–7.4). Coronal slices were obtained (350 μm thick) and incubated (>30 min) at room temperature in ACSF. Slices were then transferred to an immersion recording chamber in the presence of picrotoxin (50 μM, GABA_A_ receptor antagonist) and CGP54626 (1 μM, GABA_B_ receptor antagonist) and superfused at 2 mL/min with gassed ACSF and visualized under an Olympus BX51WI microscope (Olympus Optical, Japan).

### Electrophysiological Recordings

**Figure 1:**
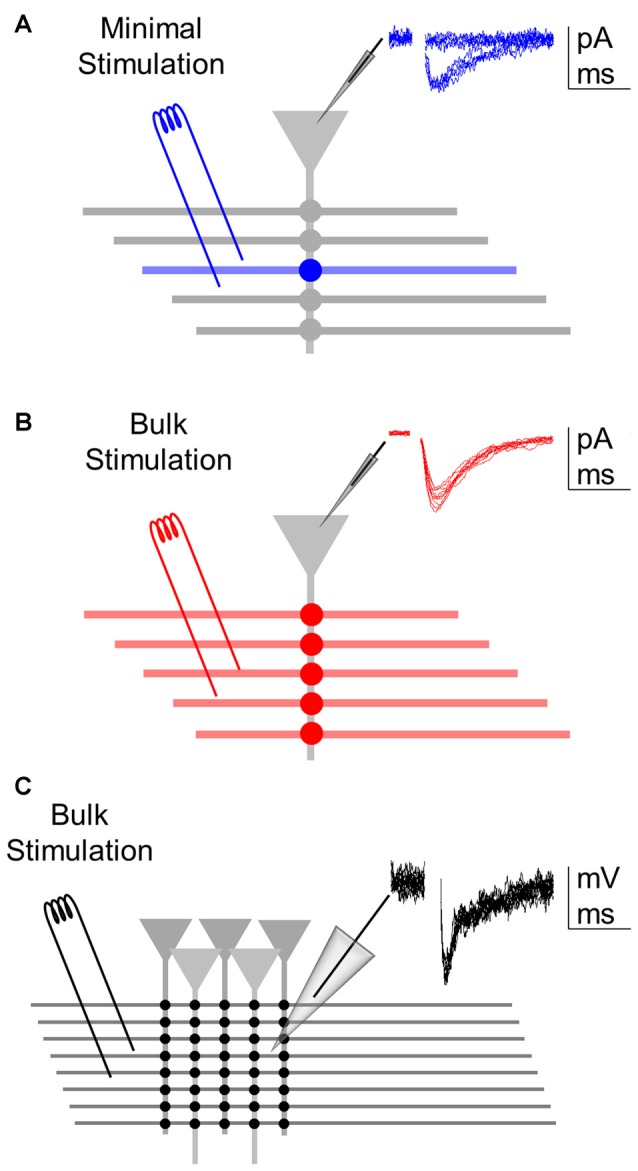
Assessing synaptic transmission at different levels of analysis. **(A)** Minimal stimulation to activate single or few axons allow recording of a single synapse (blue) from a whole-cell recorded postsynaptic neuron. Inset: representative excitatory postsynaptic currents (EPSC) recordings showing failures and successes (of relatively constant low amplitude) in synaptic transmission. **(B)** Bulk stimulation of multiple fibers to activate a synapse ensemble (red) from the whole-cell recorded postsynaptic neuron. Inset: representative excitatory postsynaptic potentials (EPSCs) showing relatively constant amplitudes void of failures. **(C)** Bulk stimulation of a synapse population (black) recorded extracellularly through local field potential. Inset: representative fEPSP recordings showing relatively constant amplitudes.

Electrophysiological recordings from CA1 pyramidal neurons were made in whole-cell configuration of the patch-clamp technique (Figures 1A,B). Patch electrodes had resistances of 3–10 MΩ when filled with an internal solution containing (in mM): KMeSO_4_ 135, KCl 10, HEPES 10, NaCl 5, ATP-Mg^+2^ 2.5 and GTP-Na^+^ 0.3 (pH = 7.3). Recordings were obtained with PC-ONE amplifiers (Dagan Instruments, MN, USA). Membrane potential was held at −70 mV, and access (11.9 ± 2.9 MΩ) and input (246.8 ± 54.0 MΩ) resistances were monitored throughout the experiment using a −5 mV pulse. Cells were discarded when series and input resistances changed >20%. Extracellular field recordings were performed using a patch pipette filled with ACSF. Field recordings were obtained with an EX-1 amplifier (Dagan Instruments, MN, USA). Signals were sent to a Pentium-based PC through a DigiData 1440A interface board. Intracellular signals were low-pass filtered at 1 KHz and extracellular signals were bandpass filtered at 3–300 Hz, both acquired at 10 KHz sampling rate. The pCLAMP 10.4 (Molecular Devices) software was used for stimulus generation, data display, acquisition and storage.

### Synaptic Stimulation

Theta capillaries filled with ACSF were used for bipolar stimulation and placed in the CA1 *striatum radiatum* to stimulate Schaffer collateral afferents from CA3. Paired pulses (0.2 ms duration with 50 ms interval) were continuously delivered at 0.33 Hz using a stimulator S-910 (Dagan Instruments) through an isolation unit. EPSCs were recorded from CA1 pyramidal neurons. Stimulus intensity (0.1–10 mA) was adjusted to meet “minimal” conditions that putatively stimulate single or very few presynaptic fibers (Figure 1A; Raastad, 1995; Isaac et al., 1996; Dobrunz and Stevens, 1997; Perea and Araque, 2007; Navarrete and Araque, 2010; Navarrete et al., 2012; Perea et al., 2016) or the “bulk” conditions that diminished the incidence of synaptic failures to 0 (Figure 1B). During bulk stimulation, fEPSPs were also recorded extracellularly (Figure 1C). Individual cells were recorded in both minimal and bulk stimulation protocols. Synaptic parameters analyzed were: probability of release (ratio between the number of successes vs. total number of stimuli); synaptic potency (mean peak amplitude of the successes) and synaptic efficacy (probability of release *times* synaptic potency; in bulk, mean peak amplitude of all responses; Raastad, 1995; Isaac et al., 1996; Perea and Araque, 2007; Navarrete and Araque, 2010; Navarrete et al., 2012; Perea et al., 2016). To illustrate time course, synaptic parameters were grouped into 1 min bins.

To stimulate the release of eCB via neuronal depolarization (ND), one CA1 pyramidal neuron was depolarized to 0 mV for 5 s (Kreitzer and Regehr, 2001a; Wilson and Nicoll, 2001; Ohno-Shosaku et al., 2002; Chevaleyre and Castillo, 2004; Navarrete and Araque, 2008, 2010; Gómez-Gonzalo et al., 2015). To determine synaptic changes after ND, 3 min before ND (basal) were compared with 2 min after the stimulus. The presence of synaptic depression or potentiation was determined in individual synapses when the synaptic efficacy obtained within 2 min after the stimuli changed more than 2 standard deviations below or above 3 min of baseline respectively for depolarized cells and cells recorded within 60–120 μm from ND. Experimental and simulation data was tested for significance using one-tailed paired *t*-tests between basal and post ND (left-tailed for DSE, right-tailed for eSP), and two-tailed for field recording tests. Significance is denoted by “*” ≡ *p* < 0.05, “**” ≡ *p* < 0.01 and “***” ≡ *p* < 0.001. Additional analyses were performed using one-sample power analysis (Chow et al., 2007).

### Mathematical Modeling

The mathematical model was developed in MATLAB to simulate synapse ensemble and synapse population experiments using a Monte Carlo method drawing from normal distributions of single synapse experimental data. Single synapse synaptic efficacy was generated by randomly selecting values from normal distributions with sample mean (*μ*) and sample standard deviation (*σ*) obtained from normalized experimental data. Baseline values were obtained from a normal distribution of baseline minimal data (*μ*_baseline_, *σ*_baseline_) (equation 1).

(1) $$P_{\text{baseline}}(x)\;=\;\frac{1}{\sigma_{\text{baseline}}\sqrt{2\pi }}e^{\frac{-{\left(x-\mu_{\text{baseline}}\right)}^{2}}{2\sigma_{\text{baseline}}^{2}}}$$

To model the effect of eCB on aggregated synapses, subsets of simulated synapses were made up of regulated and unregulated synapses. From our experimental results, we found 36% of the synapses were regulated so we generated 36% of our synapses from a normal distribution of regulated synapse experimental data of normalized synaptic efficacies after ND (*μ*_regulated_, *σ*_regulated_), and 64% of the synapses were generated from a normal distribution from unregulated synapse experimental data after ND (*μ*_unregulated_, *σ*_unregulated_) to form a bimodal distribution (equation 2). Because CA1 pyramidal neurons sum EPSCs linearly (Cash and Yuste, 1998, 1999), we modeled synapse ensemble responses by linear averaging individual drawn synapses having normalized synaptic efficacies.

(2) $$P_{\text{ecb}}(x)\;=\;\frac{0.36}{\sigma_{\text{regulated}}\sqrt{2\pi }}e^{\frac{-{\left(x-\mu_{\text{regulated}}\right)}^{2}}{2\sigma_{\text{regulated}}^{2}}}\;+\;\frac{0.64}{\sigma_{\text{unregulated}}\sqrt{2\pi }}e^{\frac{-{\left(x-\mu_{\text{unregulated}}\right)}^{2}}{2\sigma_{\text{unregulated}}^{2}}}$$

To determine the number of synapses available (*N*_active_) in bulk stimulation configuration we divided the experimental average synapse ensemble synaptic potency (47.9 pA) by the experimental average single synapse synaptic potency (9 pA) equaling ~5 active synapses. Only a subset of the total responsive synapses should be active at any given instant, and the relationship between *N*_active_ and number of total synapses (*N*_total_) is captured in the binomial distribution (equation 3). The mean of the binomial distribution (${\bar{N}}_{\text{active}}$) is defined equaling *N*_total_ times the *Pr* (0.4) (equation 4). Algebra allows us to determine the *N*_total_ from *Pr* and ${\bar{N}}_{\text{active}}$ (equation 5).

(3) $$P(N_{\text{active}})\;=\;\frac{N_{\text{total}}!}{(N_{\text{total}}\;-\;N_{\text{active}})!N_{\text{active}}!}Pr^{N_{\text{active}}}{\left(1\;-\;Pr\right)}^{N_{\text{total}}-N_{\text{active}}}$$

(4) $${\bar{N}}_{\text{active}}\;=\;Pr*N_{\text{total}}$$

(5) $$N_{\text{total}}\;=\;\frac{{\bar{N}}_{\text{active}}}{Pr}$$

Plugging our values (${\bar{N}}_{\text{active}}$ = 5, *Pr* = 0.4) into equation 5 determined our average number of *N*_total_ for bulk stimulation was 12 synapses, which was used in simulations of bulk stimulation of synapse ensembles.

In summary, the computational model was implemented as such: let us assume that each synapse *i* has an initial synaptic efficacy *X*_i_ before ND and *Y*_i_ after ND. Let us assume that the *X*_i_s are gaussian distributed with mean *μ*_baseline_ and variance $\sigma_{\text{baseline}}^{2}$ (equation 1). Likewise, the *Y*_i_s are hypothesized to be bimodally distributed from two gaussians, one of mean *μ*_regulated_ and variance $\sigma_{\text{regulated}}^{2}$ drawn 36% of the time pseudorandomly and another of mean *μ*_unregulated_ and variance $\sigma_{\text{unregulated}}^{2}$ drawn 64% of the time pseudorandomly (equation 2). Using this convention, 12 synapses’ baseline efficacies *X*_i_ were linearly averaged together to simulate one baseline synapse ensemble, and an aggregate of regulated and unregulated efficacies *Y*_i_ were linearly averaged to represent one post ND synapse ensemble. In this fashion experiments of varying sample sizes were created and tested for significance using a paired *t*-test between baseline values and regulated values.

For simplicity, field recording simulations were performed as above but by linearly averaging together 100 “cells”, each simulated as a synapse ensemble with both linearly averaged baseline and also aggregated 36% regulated and 64% unregulated synapses. Of these 100 cells, 11% of “cells” underwent synaptic depression, 33% of “cells” underwent synaptic potentiation and 56% of “cells” underwent no change. The model performed 1000 simulations for synapse ensemble data, and 100 simulations for synapse population data, and was used to recreate experimental data and to predict *n*_0.05_ ≡ the sample size required to reach statistical significance, i.e., *p* < 0.05.

## Results

To investigate the ability of detecting synapse-specific synaptic regulation at different levels of observation, we assessed the changes induced by eCB released from CA1 pyramidal neurons on CA3-CA1 synaptic transmission. We combined whole-cell recordings from CA1 pyramidal neurons and local field potentials from the *striatum radiatum* of the CA1 region. EPSCs and fEPSPs were evoked by Schaffer collateral (SC) stimulation through bulk or minimal stimulation to activate multiple or single synapses, respectively. We use the following terminology: (1) single synapses: activity of a single synapse monitored by whole-cell neuronal recording and minimal stimulation of SC; (2) multiple synapses: combined activity of individually recorded single synapses estimated from the average synaptic efficacy of all recorded single synapses (including regulated and unregulated synapses); (3) whole-cell synapse ensembles: combined simultaneous activity of several synapses into a single postsynaptic neuron; assessed from whole-cell recorded EPSCs evoked by bulk stimulation of SC; and (4) synapse population: combined activity of a large number of synapses contacting multiple postsynaptic neurons; assessed from the extracellularly recorded fEPSPs evoked by bulk stimulation of SC.

### Endocannabinoid-Induced Suppression of Excitation at Single and Multiple Synapse Levels

**Figure 2:**
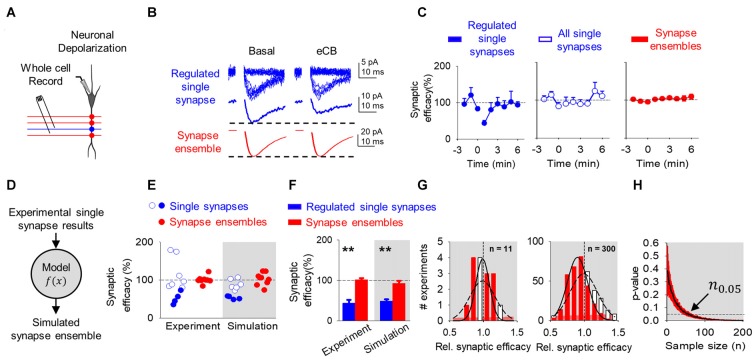
Endocannabinoid (eCB)-mediated synaptic depression can be observed at single synapses, but it is concealed at multiple synapses and synapse ensembles. **(A)** Scheme representing a whole-cell recorded CA1 pyramidal neuron and schaffer collaterals (SC) minimal stimulation. **(B)** Representative EPSC traces showing successes and failures in neurotransmitter release during minimal stimulation (blue, top traces), their average trace (blue, middle traces) and average EPSC obtained by bulk SC stimulation (red, bottom traces) before (basal) and after neuronal depolarization (ND) that stimulate eCB release (eCB). **(C)** Relative synaptic efficacy (from basal) vs. time in depressed single synapses, all single synapses, and all synapse ensembles. Zero time indicates eCB release by ND. **(D)** Monte Carlo scheme of synapse ensemble depolarization-induced suppression of excitation (DSE) using single synapse data. **(E)** Relative synaptic efficacy (from basal) after ND-evoked eCB release. Individual experimental and simulated data of regulated (blue circles) and unregulated (white circles) single synapses and synapse ensembles (red circles). **(F)** Average experimental and simulated data of regulated single synapses (blue bars) and synapse ensembles (red bars). **(G)** Histogram distributions of simulated normalized synaptic efficacy of amplitude baseline (white) with modulated (red) data for *n* = 11 (left) and *n* = 300 (right). **(H)** Computational model-predicted *p*-value vs. sample size. Data are represented as mean ± SEM. **p < 0.01.

Retrograde eCBs are known to depress hippocampal synaptic transmission by activation of presynaptic CB1Rs (Wilson and Nicoll, 2001; Ohno-Shosaku et al., 2002; Chevaleyre and Castillo, 2004; Navarrete and Araque, 2010; Araque et al., 2017). We first investigated eCB-mediated synaptic depression at the single synapse level. We recorded from a CA1 pyramidal neuron using the whole-cell configuration of the patch-clamp technique and recorded EPSCs evoked by stimulation of the SC using the minimal stimulation method, which putatively activates one or very few synapses (Raastad, 1995; Isaac et al., 1996; Dobrunz and Stevens, 1997; Perea and Araque, 2007; Navarrete and Araque, 2010; Navarrete et al., 2012; Perea et al., 2016). In this condition, synaptic responses showed failures and successes in neurotransmitter release (Raastad, 1995; Isaac et al., 1996; Dobrunz and Stevens, 1997; Perea and Araque, 2007; Navarrete and Araque, 2010; Navarrete et al., 2012; Perea et al., 2016). We quantified the probability of neurotransmitter release (Pr), the synaptic potency (i.e., the amplitude of successful EPSCs) and the synaptic efficacy (i.e., Pr times the synaptic potency). After that, we depolarized the pyramidal neuron to 0 mV for 5 s to stimulate eCB release (Wilson and Nicoll, 2001; Ohno-Shosaku et al., 2002; Chevaleyre and Castillo, 2004; Navarrete and Araque, 2010; Araque et al., 2017), and monitored the synaptic parameters at single synapses (Figure 2A).

In agreement with previous reports (Navarrete and Araque, 2010), eCB released by ND transiently depressed synaptic efficacy (from 2.9 ± 1.3 pA to 1.6 ± 0.8 pA; *n* = 4; *p* < 0.05; Figure 2B) in 36% of synapses (4 out 11). The analysis of synapses that underwent the phenomenon indicates that the synaptic depression was associated with a reduction in Pr (from 0.3 ± 0.1 to 0.2 ± 0.1; *n* = 4; *p* < 0.05) without synaptic potency changes (from 9.6 ± 3.7 pA to 8.7 ± 3.6 pA; *n* = 4; *p* = 0.10; Figures 2B,C), suggesting a presynaptic mechanism (Debanne et al., 1996). This phenomenon is consistent with the DSE phenomenon that is mediated by activation of presynaptic CB1Rs (Ohno-Shosaku et al., 2002; Chevaleyre et al., 2006).

Then, to estimate the overall effects of eCBs in multiple synapses, we pooled all the data of individual synapses, regardless of the presence or absence of DSE. In this analysis, no significant changes were observed in the synaptic efficacy (from 3.1 ± 1.9 pA to 2.5 ± 1.6 pA; *n* = 11; *p* = 0.06), Pr (from 0.3 ± 0.2 to 0.3 ± 0.2; *n* = 11; *p* = 0.17) or synaptic potency (from 9.0 ± 3.0 pA to 8.2 ± 2.9 pA; *n* = 11; *p* = 0.13; Figures 2B,C). A one-sample power analysis on the pooled synaptic efficacies at *α* = 0.05 and *β* = 0.2 predicted that it would require 180 experiments before observing significance with pooled data. This indicates that the effects on individual single synapses may be concealed when mingled with multiple synapses, and suggests that subtle regulation of specific synapses may be experimentally undetected at the scale of multiple synapses.

To experimentally test this idea, we stimulated SC using bulk stimulation to simultaneously activate a synapse ensemble on a single neuron, as indicated by the larger EPSCs and the absence of synaptic failures. In this condition, no DSE was statistically observed (EPSC amplitude before and after ND was 47.9 ± 28.4 pA to 51.8 ± 31.8 pA; *n* = 9; *p* = 0.96; Figures 2B,C), which agrees with the concealed synaptic regulation when considering together single synapses undergoing or not synaptic regulation. A one-sample power analysis on this bulk stimulation data at *α* = 0.05 and *β* = 0.2 predicted that it would require 1255 experiments before observing significance.

Observing a significant effect at the single synapse level required us to stratify the data and focus only on the specific synapses that were regulated following ND. Unfortunately, the bulk stimulation paradigm does not allow stratification of regulated and unregulated synapses from synapse ensemble recordings, and there is no straightforward way to implement power analysis on data we expect to be subtly made from a bimodal distribution of regulated and unregulated data. Because of this, we then developed a computational model based on the experimental data to recreate synapse ensemble results using a Monte Carlo method drawing from single synapse normalized synaptic efficacy experimental data of baseline (equation 1) and comparing to aggregated regulated and unregulated single synapse normalized synaptic efficacy experimental data (equation 2) (Figure 2D). Here, baseline synaptic efficacies were values before ND, regulated synaptic efficacies were those synapses whose efficacies were reduced by 2 standard deviations below baseline the 2 min following ND, and unregulated were synapses whose synaptic efficacies were not reduced below 2 standard deviations of baseline synaptic efficacies. Based on experimental data (see Figure 2C), the model considered a variable DSE amplitude displayed by 36% of single synapses, and simulated the synaptic changes occurring in regulated single synapses, unregulated single synapses and synapse ensembles (including regulated and unregulated synapses).

To estimate the number of synapses recruited in synapse ensembles, we considered that the stimulated synapses had a nonzero probability of failure, and we divided the average amplitude for bulk stimulation (47.9 pA) by the average synaptic potency of minimal stimulation experiments (9.0 pA), which indicates the simultaneous activation of 5 synapses. While bulk stimulation activated on average 5 synapses simultaneously (${\bar{N}}_{\text{active}}$), activated synapses would shuffle between a larger pool of receptive presynaptic terminals (*N*_total_) that occasionally fail to respond. This arrangement is defined by the binomial distribution (equation 3), which we used the mean of the binomial distribution and our values for ${\bar{N}}_{\text{active}}$ = 5 and *Pr* = 0.4 from all DSE and eSP minimal experiments to determine *N*_total_ of 12 synapses on average are available during our bulk stimulation (equation 4 and 5). Synapse ensembles were simulated by linearly averaging together 12 single synapses of 36% regulated (52 ± 16.3%) and 64% unregulated (116 ± 43%) and compared with ensembles made from baseline synapses (100 ± 61%). The computational program faithfully reproduced the DSE observed at single synapses in both regulated (Figures 2E,F, blue circles) and unregulated (Figures 2E,F, white open circles) single synapses and synapse ensembles (Figures 2E,F, red circles). These results suggest that the observed DSE in a subset of single synapses can be statistically undetected when unregulated synapses are included or when they are recorded together in synapse ensembles.

Because the phenomenon was observed by examining individual synapses but was concealed when pooling together regulated and unregulated single synapses or examining synapse ensembles, it indicates that the concealment is not a biologic phenomenon, rather it is due to statistical limitations. We then hypothesized that this phenomenon could be revealed by enhancing the statistical power through increasing the sample size. To test this, we used the Monte Carlo computational approach to model the synaptic changes induced by DSE at mingled synapse ensembles and to estimate the impact of the sample size (*n*) on the statistical significance of those changes measured by the *p*-value between baseline and modulated simulations. The model shows that the distribution of synapse ensemble DSE modulation is unresolved from the baseline distribution at *n* = 11, however the distribution of DSE modulation at *n* = 300 allows us to see a difference between the regulated and baseline distributions at more reduced synaptic efficacies (Figure 2G). Furthermore, considering the experimentally observed DSE amplitude (52.3 ± 16.3%) in 36% of single synapses, the simulations of synapse ensembles predicted that at least 56 experiments are required to detect a statistically significant difference (*n*_0.05_ = 56; *p* < 0.05) in the synaptic efficacy (Figure 2H).

### Endocannabinoid-Induced Astrocyte-Mediated Synaptic Potentiation at Single and Multiple Synapse Levels

**Figure 3:**
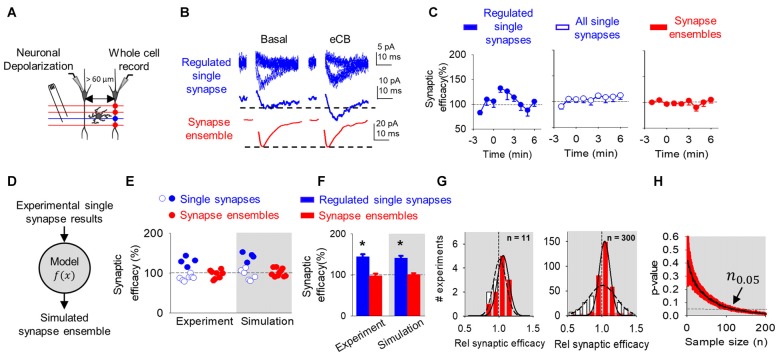
eCB-mediated synaptic potentiation can be observed at single synapses, but it is concealed at multiple synapses and synapse ensembles. **(A)** Scheme representing paired whole-cell recorded CA1 pyramidal neurons and SC minimal stimulation. **(B)** Representative EPSC traces showing successes and failures in neurotransmitter release during minimal stimulation (blue, top traces), their average trace (blue, middle traces) and average EPSC obtained by bulk SC stimulation (red, bottom traces) before (basal) and after ND that stimulate eCB release (eCB). **(C)** Relative synaptic efficacy (from basal) vs. time in potentiated single synapses, all single synapses and all synapse ensembles. Zero time indicates eCB release by ND. **(D)** Monte Carlo scheme of synapse ensemble eSP using single synapse data. **(E)** Relative synaptic efficacy (from basal) after ND-evoked eCB release. Individual experimental and simulated data of regulated (blue circles) and unregulated (white circles) single synapses and synapse ensembles (red circles). **(F)** Average experimental and simulated data of regulated single synapses (blue bars) and synapse ensembles (red bars). **(G)** Histogram distributions of simulated normalized synaptic efficacy of baseline (white) with modulated (red) data for *n* = 11 (left) and *n* = 300 (right). **(H)** Computational model-predicted *p*-value vs. sample size. Data are represented as mean ± SEM. *p < 0.05.

Released eCB during ND has been shown to indirectly regulate synaptic transmission and plasticity through activation of astrocytes in hippocampus, cortex and striatum (Navarrete and Araque, 2010; Min and Nevian, 2012; Gómez-Gonzalo et al., 2015; Martín et al., 2015; Andrade-Talavera et al., 2016). In the hippocampus, activation of astrocytic CB1Rs stimulate the release of glutamate, which activates presynaptic type I mGluRs and induces the synaptic potentiation (eSP) of single synapses in relatively distant neurons (between 60 μm and 120 μm away from the stimulated neuron; Navarrete and Araque, 2010), a phenomenon termed lateral synaptic regulation (Covelo and Araque, 2016; Araque et al., 2017). We then investigated this phenomenon at the single synapse level. We performed paired recordings from two CA1 pyramidal neurons while recording EPSCs evoked by SC stimulation using the minimal stimulation method to activate single synapses (Raastad, 1995; Isaac et al., 1996; Dobrunz and Stevens, 1997; Perea and Araque, 2007; Navarrete and Araque, 2010; Navarrete et al., 2012; Perea et al., 2016). We depolarized one pyramidal neuron (termed homoneuron) to 0 mV for 5 s to stimulate eCB release (Wilson and Nicoll, 2001; Ohno-Shosaku et al., 2002; Chevaleyre and Castillo, 2004; Navarrete and Araque, 2010; Araque et al., 2017), and monitored synaptic parameters at single synapses in an adjacent neuron (termed heteroneuron) located 60–120 μm away from the depolarized neuron (Figure 3A; Navarrete and Araque, 2010; Gómez-Gonzalo et al., 2015).

Consistent with previous reports (Navarrete and Araque, 2010; Gómez-Gonzalo et al., 2015), the ND enhanced synaptic efficacy (from 4.6 ± 2.4 pA to 5.8 ± 2.5 pA; *n* = 4; *p* < 0.01) in 36% (4 out of 11) of single synapses recorded in the non-stimulated heteroneuron (Figures 3B,C). This synaptic potentiation was associated with an increase in Pr (from 0.5 ± 0.1 to 0.6 ± 0.1; *n* = 4; *p* < 0.01) without changes in synaptic potency (from 8.9 ± 4.1 pA to 9.2 ± 4.0 pA; *n* = 4; *p* = 0.06), again suggesting a presynaptic mechanism (Navarrete and Araque, 2010; Gómez-Gonzalo et al., 2015). Like DSE, pooling together all the recorded synapses (i.e., regulated and unregulated synapses), no statistical changes were observed in the synaptic efficacy (from 4.8 ± 2.8 pA to 5.0 ± 2.9 pA; *n* = 11; *p* = 0.29), Pr (from 0.5 ± 0.1 to 0.5 ± 0.2; *n* = 11; *p* = 0.11) or synaptic potency (from 10.3 ± 5.2 pA to 9.8 ± 4.0 pA; *n* = 11; *p* = 0.22; Figures 3B,C). A one-sample power analysis on the pooled synaptic efficacies at *α* = 0.05 and *β* = 0.2 predicted that it would require 4265 experiments before observing significance. We further recorded synapse ensemble activity using SC bulk stimulation. In this condition, no significant changes were observed in the EPSC amplitude before and after ND (from 46.4 ± 20.9 pA to 43.5 ± 20.0 pA; *n* = 8; *p* = 0.96; Figures 3B,C). A one-sample power analysis on the pooled bulk stimulation data at *α* = 0.05 and *β* = 0.2 predicted that it would require 1045 experiments before observing significance. Taken together, these results indicate that the synaptic regulation of a subset of individual synapses can be detected at single synapse level of analysis, but become veiled by the activity of multiple synapses analyzed or recorded together.

To explain the differences found between the scales of observation, we used the mathematical model to simulate synapse ensemble experiments. We used a Monte Carlo method drawing from single synapse experimental results (baseline: 100 ± 58%; regulated: 130 ± 12%; unregulated: 89 ± 8%) to calculate merged regulated and unregulated synapse ensemble data (Figure 3D). The model reproduced regulated, unregulated single synapse, and synapse ensemble experimental data (Figures 3E,F). Baseline and eSP modulated synapse ensemble distributions appeared similar at *n* = 11 and deviated at *n* = 300 (Figure 3G). Comparing these distributions as sample size increased, the model predicted that over 113 experiments should be performed to reach a significant difference (*n*_0.05_ = 113; *p* < 0.05) following ND at the synapse ensemble level (Figure 3H).

### Endocannabinoid Modulation at Synapse Population Level Observed Through Field Recordings

**Figure 4:**
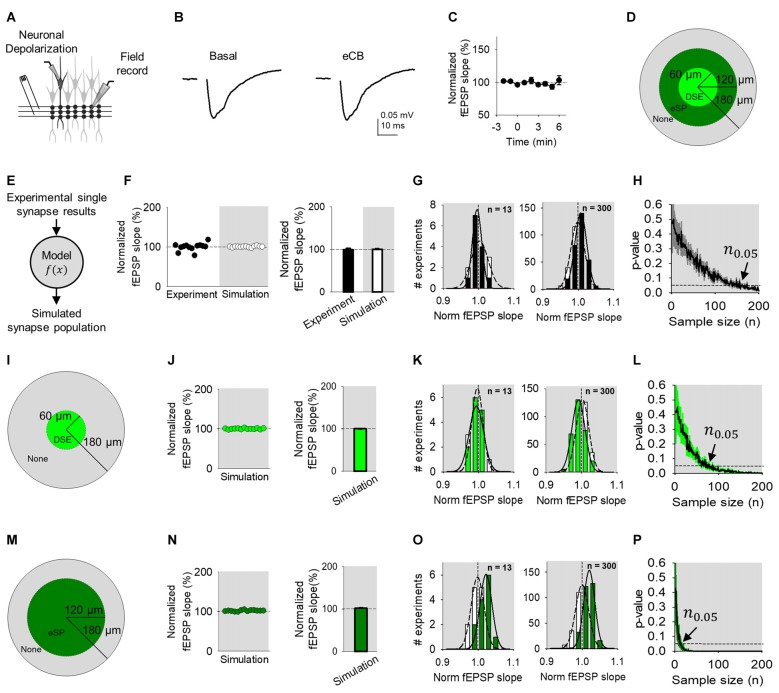
eCB-mediated synaptic regulation is undetected in Field recordings. **(A)** Scheme representing a whole-cell recorded CA1 pyramidal neuron, extracellular electrode recording fEPSPs and SC bulk stimulation. **(B)** Average fEPSP traces before (basal) and after ND of the whole-cell recorded neuron to stimulate eCB release (eCB). **(C)** Relative fEPSP slope (from basal) vs. time. Zero time indicates eCB release by ND. **(D)** Modulation topology of synapse population. **(E)** Monte Carlo scheme of synapse population using single synapse data. **(F)** Individual experimental and simulated data of relative fEPSP slope (from basal) after ND-evoked eCB release. **(G)** Simulated distributions normalized slope of baseline (white) with modulated (black) data for *n* = 13 (left) and *n* = 300 (right). **(H)** Computational model-predicted *p*-value vs. sample size. **(I)** Modulation topology of synapses undergoing DSE-only. **(J)** Simulated fEPSP slope data of DSE-only. **(K)** Simulation distributions of DSE-only normalized slope baseline (white) and modulated (green) data for *n* = 13 (left) and *n* = 300 (right). **(L)** Computational model-predicted *p*-value vs. sample size for synapses undergoing DSE-only. **(M–P)** As **(I–L)**, but for synapses undergoing eSP-only. Data are represented as mean ± SEM.

Next, we assessed how the regulation of a subset of single synapses would impact a large synapse population. We depolarized a CA1 pyramidal neuron to stimulate eCB release while extracellularly recording fEPSPs evoked by SC bulk stimulation (Figure 4A). No significant changes were found in the normalized fEPSP slope following ND (from 96 ± 7.0 to 101.3 ± 12.1; *n* = 13; *p* = 0.94; Figures 4B,C). ND-induced eCB signaling is known to depresses synaptic transmission in the stimulated neuron and relatively nearby neurons (≤60 μm away from the depolarized neuron), potentiate synaptic transmission indirectly through astrocytes in relatively more distant cells (60–120 μm away from the depolarized neuron), and leave synapses unaffected beyond 120 μm away from the eCB source (Figure 4D; Navarrete and Araque, 2010; Araque et al., 2017). Previous reports applying voltage sensitive dyes or calcium imaging techniques to SC stimulation described areas of activity in CA1 of 400 μm in diameter (MacVicar and Hochman, 1991; Nagai et al., 2004). Therefore, the proposed topology of our field recordings and subsequent simulations was represented by cells spread across a circular surface with radius of 180 μm (Figure 4D).

We utilized the model to reproduce our synapse population experimental findings using a Monte Carlo method drawing from regulated and unregulated single synapse experimental data (Figure 4E). For simplicity, the model linearly averaged together simulations of 100 cells, each composed of synapse ensemble simulations encompassing 12 grouped synapses of regulated and unregulated synapses. Of the population modeled, proximal cells within 60 μm (11%) had 36% of synapses that underwent synaptic depression, from 60 μm to 120 μm (33%) distal cells housed 36% of synapses that received synaptic potentiation and from 120 μm to 180 μm (56%) no eCB short-term plasticity occurred (Figure 4D). Computational simulations recreated the results found using local field recordings (Figure 4F). Comparing distributions of simulated baseline and regulated normalized fEPSP slope, a rightward shift occurred, denoting a potentiation when *n* = 300 that was hidden at *n* = 13 (Figure 4G). Further, the model predicted a sample size of at least 139 experiments is required to reveal a statistically significant effect (*n*_0.05_ 139; *p* < 0.05) at the population level (Figure 4H).

To computationally untangle the opposing effects of DSE and eSP in the synapse population, we simulated these phenomena separately. First, modeling only-DSE field recordings (Figure 4I), still created similar results to experimental data (Figure 4J). Simulated distributions of DSE regulation of the synapse population was overlapping to baseline distributions at *n* = 13, however, the two were distinguishable at *n* = 300 (Figure 4K). The model found statistically significant differences of DSE-only field recordings with sample sizes of 61 experiments (*n*_0.05_ = 61; *p* < 0.05; Figure 4L). Next, simulation of synapse populations of eSP-only (Figure 4M) also reproduced the experimental data (Figure 4N). However, the differences between simulated eSP-only and baseline distributions began to appear in *n* = 13 and *n* = 300 (Figure 4O). Computationally increasing the sample size predicted that field recordings of eSP-only modulation would require only 12 experiments to detect statistically significant results (*n*_0.05_ = 12; *p* < 0.05; Figure 4P). Interestingly, these results indicate that eSP of synapse populations might be feasibly detected experimentally using field recordings if present in isolation. However, because the experimental presence of DSE that opposes eSP, field recordings are the least effective of the three methods tested to observe statistically significant effects on eCB-mediated synaptic regulation.

**Figure 5:**
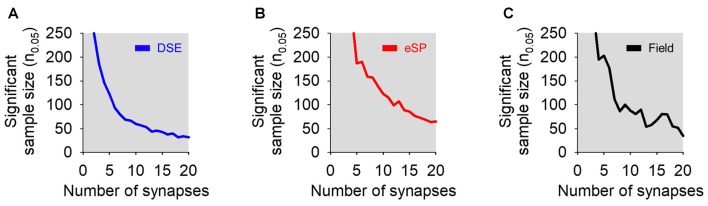
Effects of varying the number of total simulated synapses in bulk stimulation conditions on statistical significance. **(A)** Sample size required to reach statistical significance (i.e., *p* < 0.05) vs. number of total synapses in simulated DSE (blue) in bulk stimulation whole-cell recorded experiments. **(B)** As in **(A)**, but in simulated eSP (red) instead DSE. **(C)** Simulated field recording experiments of a synapse population comprised of cells undergoing DSE or eSP with variable number of total synapses (black).

We were then interested in how modeled predictions changed with adjustments to the number of synapses available on cells during bulk stimulation (*N*_total_). To answer this, we performed simulations of DSE, eSP and field experiments to determine *n*_0.05_ while varying *N*_total_ from 1 to 20 synapses. In all three simulations, increasing *N*_total_ led to a smaller sample size to observe statistical significance of bulk stimulation experiments, either using whole-cell or field recordings (Figure 5). It should be noted that stimulating up to 20 synapses on a single cell still required a large sample size to reach *n*_0.05_.

Taken together these results indicate that synapse-specific regulation may be observed at single synapse level of analysis, but it may be experimentally concealed at large observational levels, unless results from a relatively large sample size are considered.

## Discussion

**Figure 6:**
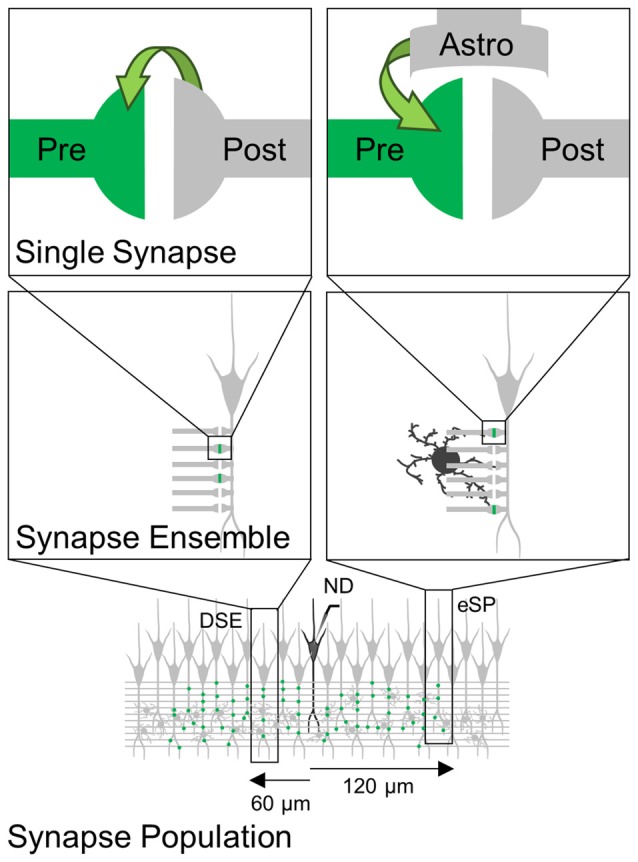
Scheme representing eCB-induced synaptic regulation at different levels of observation. eCBs released by ND induces synapse-specific modulation (green) through direct activation of cannabinoid receptors type 1 (CB1Rs) at presynaptic terminals (left) or through indirect activation of astrocytes (right). These synapse-specific phenomena observed at single synapses are concealed when monitoring synapse ensemble or synapse population.

We have investigated the extent to which eCB-mediated short-term synaptic plasticity scaled up from the single synapse level to the synapse ensemble and synapse population, and whether the regulatory effects can be experimentally detected at different levels of observation. Consistent with previous results using minimal stimulation (Navarrete and Araque, 2010; Gómez-Gonzalo et al., 2015) upon ND that stimulate the release of eCBs, a subset of synapses on depolarized cells undergo a synaptic depression known as DSE, while synapses on cells distally located (>60 μm away from the eCB source) to the depolarized neuron experienced an astrocyte-mediated synaptic potentiation. At both distances, stratifying the single synapse data was required to observe a statistically significant difference in synaptic efficacy following ND, and this effect was concealed when regulated synapses were averaged with unregulated synapses. These results suggest that eCB-induced synaptic modulation is synapse-specific whether mediated by neurons or astrocytes, and that the level of observation determines the ability to experimentally detect the phenomena (Figure 6).

Synapse ensemble activity from a single neuron recorded using bulk stimulation also failed to display either DSE or eSP in proximal or distal synapse ensembles, respectively. Since synapse ensembles were recorded from the same cells as regulated single synapses, our results suggest that eCB signaling does not impact all synapses within one neuron, but instead, eCB-mediated short-term synaptic plasticity targets specific synapses. This is also in line with our single synapse experiments requiring the data to be stratified to observe a significant change. Using a Monte Carlo approach to mathematically simulate bulk stimulation results recreated experimental data for both single synapse and synapse ensemble results. Modeling findings suggested that eCB synapse-specific modulation was concealed in synapse ensembles confirming the experimental data and further predicted that both phenomena would be exposed after a larger experimental sample size (*n*_0.05_ = 56 for DSE and *n*_0.05_ = 113 for eSP).

Experiments using extracellular field recordings also failed to measure a statistically significant effect of the eCB synapse-specific regulation. Using a Monte Carlo approach again, we reproduced the experimental synapse population results by drawing from single synapse experimental data. The model then predicted that a higher sample size was necessary to observe a statistically significant effect (*n*_0.05_ = 139). Simulations incorporating only DSE or eSP of synapse populations predicted significance in smaller sample sizes (*n*_0.05_ = 61 for DSE-only and *n*_0.05_ = 12 for eSP-only). Our model suggests a lack of fidelity in field recordings may be due to blending the influence of a mass of synapses that do not house modulated synapses. In addition, particular to eCB modulation itself was the topology of synaptic depression in proximal synapses to eCB source counteracting synaptic potentiation at more distal synapses. Astrocyte mediated potentiation (eSP) has been shown to occur at nearby synapses to ND in the presence of the G_i/o_ protein antagonist pertussis toxin to block CB1R signaling in neurons (DSE), however in normal conditions DSE overcomes eSP in nearby synapses (Navarrete and Araque, 2010).

The present study aims to comment on the subtle nature of synapse-specific regulation, and the proper methods of recording these phenomena. Ordinarily bulk stimulation is adequate to observe broad synaptic plasticity, however regulation via eCB subtly affects only a subset of synapses, requiring more accurate measurements. Synapse specificity allows neurons to form detailed subcellular targets with surrounding cells to encode multiple representations with many degrees of freedom (Buzsáki, 2010), and the above results test our ability to resolve these interactions. Memory formation relies on plasticity occurring on a fine scale of specific synapses in a network of cells. eCB synaptic regulation affects a small subset of synapses and is subtle to experimenters, but their effect is significant in proper network functioning (Bernard et al., 2005).

Depolarization-induced suppression of inhibition (DSI) was initially observed in the cerebellum and hippocampus (Llano et al., 1991; Pitler and Alger, 1992). However, the existence of hippocampal DSE has been controversial since its discovery in the cerebellum (Kreitzer and Regehr, 2001b). Later studies showed a lessening effect of hippocampal DSE compared to DSI and that hippocampal DSE requires at least a 7 s depolarization to have a significant effect on synaptic efficacy when bulk stimulation methods are used (Ohno-Shosaku et al., 2002). Present results may explain the controversies regarding the existence of hippocampal DSE. While DSE was not observed when multiple synapses are bulk stimulated and recorded by whole-cell or local field potentials, DSE become conspicuous at the single synapse scale only, as shown in this study and others (Navarrete and Araque, 2010; Gómez-Gonzalo et al., 2015). At the heart of the hippocampal DSE controversy may be that unlike DSI, DSE may be a synapse-specific phenomenon in the hippocampus requiring minimal stimulation and stratification of data to be identified.

Astrocytes regulate synaptic activity through their participation in the tripartite synapse and the release of gliotransmitters (Araque et al., 1999, 2014; Volterra and Meldolesi, 2005). Although astrocyte-to-neuron signaling has been challenged previously (Petravicz et al., 2008; Nedergaard and Verkhratsky, 2012; Ding et al., 2013), astrocyte-mediated synaptic regulation is supported by accumulating evidence (Pascual et al., 2005; Perea and Araque, 2007; Henneberger et al., 2010; Di Castro et al., 2011; Panatier et al., 2011; Min and Nevian, 2012; Gómez-Gonzalo et al., 2015, 2017; Martín et al., 2015; Scofield et al., 2015; Andrade-Talavera et al., 2016; Perea et al., 2016). Moreover, astrocyte synaptic regulation has been shown to be synapse-specific, suggesting that astrocyte signaling does not exert broad unspecific effects, but rather subtle and fine regulatory phenomena on specific synapses (Navarrete and Araque, 2010; Gómez-Gonzalo et al., 2015; Martín et al., 2015). Present results show that these fine synaptic regulatory phenomena can be concealed at large observation scales, such as bulk stimulation or field potential recordings.

Numerous studies have demonstrated that synaptic regulatory phenomena can be detected at large observational scales. For instance, long term potentiation can be easily observed using local field potential recordings (Bliss and Collingridge, 1993). However, subtle mechanisms have been further identified when inspecting single synaptic interactions (Malinow and Tsien, 1990; Liao et al., 1995; Isaac et al., 1996). Likewise, while astroglial contribution to long-term synaptic plasticity has been detected when astrocyte signaling involves a large number of synapses (Henneberger et al., 2010), present results show that field recordings were unable to illuminate synapse-specific interactions that were observed at finer resolution. Therefore, caution should be exercised regarding the limitations of the techniques used to investigate synaptic regulatory mechanisms that may occur at subsets of specific synapses.

In conclusion, present experimental data and mathematical modeling show that detection of neurotransmission modulation can be strongly affected by the methodology used to assess synaptic activity, and that subtle synapse-specific regulatory phenomena, which may have important implications on the spatial extension of signaling mechanisms underlying synaptic plasticity, requires a fine level of analysis.

## Author Contributions

AA, JL and AC contributed to project conception, project design and manuscript writing. AC and RG collected and processed electrophysiology data and JL and LL developed mathematical model and ran simulations. JL and AC contributed equally to the work.

## Conflict of Interest Statement

The authors declare that the research was conducted in the absence of any commercial or financial relationships that could be construed as a potential conflict of interest.
